# The change in plasma D-dimer does not help to guide the timing of reimplantation in two stage exchange for periprosthetic joint infection

**DOI:** 10.1038/s41598-021-86890-z

**Published:** 2021-04-01

**Authors:** Thomas Ackmann, Jan Schwarze, Georg Gosheger, Tom Schmidt-Braekling, Kristian Nikolaus Schneider, Ralf Dieckmann, Sebastian Klingebiel, Burkhard Moellenbeck, Christoph Theil

**Affiliations:** 1 Department of Orthopedics and Tumor Orthopedics, Muenster University Hospital, Muenster, Albert-Schweitzer-Campus 1, 48149 Muenster, Germany; 2 Department of Orthopedic Surgery, Barmherzige Brüder Hospital, 54292 Trier, Germany

**Keywords:** Biomarkers, Diagnostic markers

## Abstract

D-dimer has been included in the criteria by the Musculoskeletal Infection Society in 2018 as a novel parameter to diagnose prosthetic joint infection (PJI). However, it is unclear how D-dimer levels change in between stages of a two-stage exchange. We prospectively investigated 30 patients who underwent a two-stage exchange using a spacer for PJI. D-Dimer, CRP and IL-6 were collected before first and second stage surgery and the difference (Δ) in between stages was calculated. The levels of plasma D-Dimer did not change from first to second stage surgery (2770 ng/ml (IQR, 1600–3770 ng/ml) versus 2340 ng/ml (IQR, 1270–4100 ng/ml); *p* = 0.8) while CRP (4.0 mg/dl (IQR, 1.7–5.5 mg/dl) versus 0.6 mg/dl (IQR, 0.5–0.8 mg/dl); *p* < 0.001) and IL-6 (21 pg/ml (IQR, 10–29 pg/ml) versus 6 pg/ml (4–9 pg/ml); *p* < 0.001) decreased. The ΔD-dimer between both stages was 300 ng/ml (range: − 2820 to 4280 ng/ml), the median ΔCRP was − 3.4 mg/dl (IQR, − 1.2 to − 4.8 mg/dl) and ΔIL-6 was − 13 pg/ml (IQR, − 4 to − 20 pg/ml). In 15 of 30 cases (50%) the D-dimer level increased between both stages, whereas the level of CRP (93%; 28/30) and IL-6 (96%; 28/29) decreased in most patients. As the level of serum D-dimers varies greatly, lacks a uniform decrease and does not identify persisting infection, surgeons should be cautious when using it at the timing of reimplantation.

## Introduction

Prosthetic joint infection (PJI) is a serious complication that can occur after total joint arthroplasty (TJA). While there are different surgical approaches depending on various clinical factors^1^, two-stage revision with staged, subsequent reimplantation is widely considered the reference standard for treatment of chronic PJIs^2,3^. During the first stage all components are removed, a thorough synovectomy and debridement of the infected tissue is performed. Usually a static or an articulating antibiotic-eluting cement spacer is inserted followed by systemic antibiotic treatment. In a planned second stage surgery after the infection is considered eradicated, the spacer is removed and a revision prosthesis is implanted. The long-term success of a two-stage exchange in eradicating PJI can vary greatly and persisting infection or reinfection is frequent^4,5^.

The diagnosis of persisting infection prior to second stage is difficult and there is no optimal test that indicates persistence of infection^6^. Surgeons often use a combination of inflammatory serum biomarkers such C-reactive protein (CRP) or interleukin-6 (IL-6), synovial markers, microbiological findings and clinical factors such as wound healing to assess the resolution of infection and to guide the timing of second stage reimplantation^6,7^. However, even most reliable tests are often inadequate to identify persistent, subclinical infection after the first stage of a two-stage revision procedure^6^. Lee et al. found a pooled sensitivity of 53% for CRP and a pooled specificity of 60% for erythrocyte sedimentation rate (ESR) in a recent review article concluding that currently no ideal parameter exists that can reliably time reimplantation^6^.

Serum D-dimer is a novel parameter that has been included in the criteria by the Musculoskeletal Infection Society (MSIS) in 2018^8^ and the findings from International consensus meeting (ICM)^9^ for the diagnosis of PJI. Several studies have investigated the diagnostic accuracy of D-dimer in distinguishing aseptic complication and PJI with conflicting results and a reported sensitivity of 38–96%^10–12^ and a specificity of 32–94%^10,11,13^. The value of D-dimer prior to reimplantation in a two-stage exchange has only been investigated sporadically with one study recommending its use^14^ and one^15^ finding a limited accuracy. Furthermore, the change of D-dimer from first stage to second stage surgery in individual patients has not been investigated although the change of a marker might be a useful indicator in diagnosing persisting infection^16^.

Therefore this study investigates the course of D-dimer, CRP and IL-6 in between stages of a planned two-stage exchange for chronic hip or knee PJI.

## Patients and methods

This prospective investigation was approved by local ethics committee of the authors’ institution (ethics committee of the University of Muenster; ref. no. 2019–666-f-S), and the study was registered in the German Clinical Trials Register (registration number: DRKS00021038). The study was conducted according to the principles of the World Medical Association Declaration of Helsinki, and written consent was obtained from all the participants.

Patients were included if chronic PJI was diagnosed in accordance with the MSIS criteria of 2018^8^. Therefore we used clinical findings such as fistula or visible implant, serum parameters synovial fluid findings (leukocyte count and percentage of neutrophiles) as well as microbiological findings from joint aspiration in order to establish the diagnosis. Synovial fluid was cultured for a minimum of 14 days on Columbia blood agar, chocolate agar and Schaedler agar.

**Figure 1:**
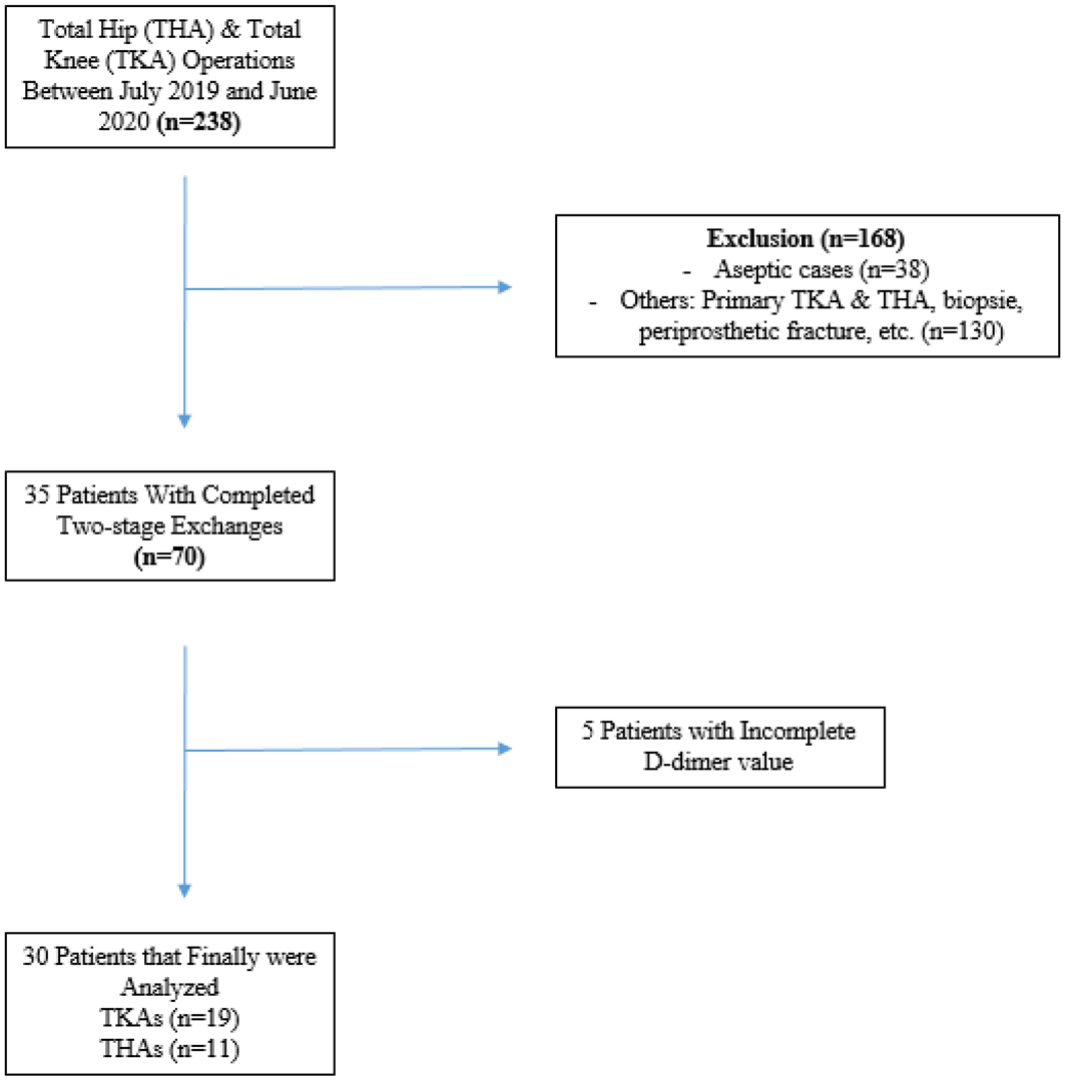
STROBE (Strengthening the reporting of observational studies in epidemiology) diagram of patients shows the study design.

In addition to serum CRP, serum IL-6 and serum D-dimer were measured before each stage. IL-6 (pg/ml) was determined by electrochemiluminescence immunoassays on the cobas e 801 chemistry analyser (Roche Diagnostics GmbH, F. Hoffmann-La Roche, Ltd., Mannheim, Germany) and D-dimer (mg/l) in citrate plasma by the STA-Liatest D-Di Plus (fibrinogen equivalent units), also by an immunoturbidimetric assay, on the STA R Max haemostasis analyser (Stago, Île-de-France, France). The change in the respective serum marker was calculating subtracting the value prior to first stage surgery from the value prior to reimplantation (an expected drop with a negative sign). Furthermore, a calculated threshold of 2850 ng/ml from a previous study using the same laboratory kit in patients undergoing revision arthroplasty was evaluated^10^. During the study period (July 2019 to June 2020) we performed complete two stage revision in 35 patients and in 30 patients preoperative D-dimers were taken at both stages (STROBE diagram, Fig. 1).

The protocol for the first-stage procedure involved removing the prosthesis and cement, taking a minimum of five tissue samples for microbiology cultures, thorough synovectomy, debridement of the infected tissue and placement of a static handmade antibiotic-eluting polymethylmethacrylate (PMMA) cement spacer (Heraeus Medical, Wehrheim, Germany) that were stabilized with intramedullary rods for knees and a handmade articulating spacer for hips. The PMMA spacers were loaded with gentamycin and clindamycin (G + C) or gentamycin, clindamycin and vancomycin (G + C + V) depending on the microbiological findings from joint aspiration. All taken tissue samples were cultured for a minimum of 14 days on Columbia blood agar, chocolate agar and Schaedler agar. All patients received at least 14 days of intravenous tailored antimicrobial treatment followed by at least four weeks oral antibiotics (for culture results see Table 1).

**Table 1:** List of the identified bacterium in the first stage revision.

Culture organism	Frequency
*Pseudomonas aeruginosa*	3% (1/30)
Staphylococcus *epidermidis*	23% (7/30)
*Cutibacterium species*	3% (1/30)
Staphylococcus *haemolyticus*	3% (1/30)
Methicillin-sensitive Staphylococcus *aureus*	10% (3/30)
Staphylococcus *lugdunensis*	3% (1/30)
*Escherichia coli*	3% (1/30)
*Moraxella species*	3% (1/30)
Streptococcus *pyogenes*	3% (1/30)
Streptococcus *dysgalactiae*	3% (1/30)
Gram negative rods	3% (1/30)
Culture-negative infection	3% (1/30)
Enterobacter *cloacae*	3% (1/30)
Enterococcus *faecium*	3% (1/30)
Staphylococcus *capitis*	13% (4/30)
Polymicrobial	13% (4/30)

Between both stages all patients received a preventive chemical thromboembolic prophylactic treatment using enoxaparin subcutaneously adapted to patient weight and renal function which was initially monitored during the in-hospital stay using factor Xa activity. After an “antibiotic holiday” of 10–14 days all patients were reassessed in an outpatient setting performing physical examination of the affected joint and analysis of CRP, Il-6 and D-dimers. An aspiration of the indwelling spacer was not performed due to expected poor diagnostic accuracy^17^. If wounds were healed and CRP and Il-6 had trended down compared to the pre-first stage results, resolution of the infection was assumed. During second-stage procedure the PMMA spacer was removed, at least five tissue samples for microbiology cultures were obtained, thorough synovectomy and debridement of the surrounding tissue was conducted and a revision implant was placed. The choice of implant was based on the extent of the bone defect and surgeon’s preference. Generally, for revision THA uncemented diaphyseal anchorage stems were combined with a cemented or cementless cup that was augmented using porous metal wedges or cages when necessary. Revision TKA was performed using a constrained or rotating hinge stemmed implant that had a cemented tibial plateau and femoral shield while the stems were usually uncemented.

Following reimplantation all patients received 14 days of intravenous antimicrobial treatment until final cultures came back negative. Persistent infection was defined by at least two positive cultures from the samples taken during the second-stage procedure before insertion of the new prosthesis. In this case oral antibiotic therapy was continued for another four weeks if wounds had healed, otherwise revision surgery with exchange of the mobile parts was performed although this was not necessary in the study cohort.

The patients’ demographic details were recorded in an electronic database. Patients who were considered to currently exhibit a systemic inflammatory response including those who had surgery within the last 3 weeks, those with inflammatory diseases of other organs such as urinary tract infection or pneumonia, patients with malignant tumors or hematologic malignancy, rheumatoid arthritis and those with history of hypercoagulation disorders did not undergo determination of D-dimer levels and were therefore excluded from the study.

### Statistical analysis

Statistical analysis of pseudonymized patient data was performed with Excel (Microsoft Corporation, Redmont, Washington, USA) and Statistical Package for the Social Sciences Statistics for Windows version 25 (IBM Corporation, Armonk, NY, USA). The Kolmogorov–Smirnov test and descriptive statistics were used to analyze distribution of data. The means and ranges were calculated for parametric data; the medians and 25–75% interquartile ranges (IQRs) were obtained for non-parametric data. The non-parametric analyses were performed using the Wilcoxon rank sum test and Mann–Whitney U-test. Frequencies are given for categorical variables and were compared in contingency tables using the chi-squared test. Statistical significance was defined as *p* ≤ 0.05. There was one missing value for interleukin-6 prior to surgery whereas all patients had D-dimers and CRP.

## Results

We included 30 patients (18 male, 12 female, 11 hips, 19 knees) that underwent complete two-stage exchange in our department. The mean age of our cohort was 70.5 years (range: 28–97 years), the mean body mass index was 30.7 kg/m^2^ (range: 21.9–48.4 kg/m^2^) and the mean days between both stages were 85.6 days (range 56–177 days). Because of the corona pandemic the time till replantation was in some cases longer than planned.

The median serum D-dimer level prior to first stage explantation and spacer implantation (2770 ng/ml (IQR, 1600–3770 ng/ml)) was not significantly different compared to the median D-dimer level prior to reimplantation (2340 ng/ml (IQR: 1270–4100 ng/ml), *p* = 0.8). While the median concentrations of CRP (4.0 mg/dl (IQR, 1.7–5.5 mg/dl) versus 0.6 mg/dl (IQR, 0.5–0.8 mg/dl), *p* < 0.001) and IL-6 (21 pg/ml (IQR, 10–29 pg/ml) versus 6 pg/ml (IQR, 4–9 pg/ml), *p* < 0.001) were significantly higher prior to first stage surgery compared to the planned second stage reimplantation. 43% of patients (13/30) had a D-dimer above 2850 ng/ml prior to reimplantation which was the calculated threshold in the diagnosis of PJI at our institutional laboratory in patients undergoing revision arthroplasty^10^.

In 15 of 30 cases (50%) the D-dimer level increased between the first and second stage surgeries and decreased in the other 15 cases. Because of this high variability we investigated potential differences in demographic characteristic and found that patients who exhibited an increase in plasma D-dimer levels had a higher median BMI, were more likely to have total hip arthroplasty infection and a longer period in between stages. However with the numbers available, there was no statistical difference. The value of CRP and IL-6 between stages decreased in 93% (28/30) and 96% (28/29) of patients, respectively.

The mean delta D-dimer between both stages was 300 ng/ml (range: − 2820 to 4280 ng/ml) or 3% increase (IQR 46% decrease to 58% increase). The median delta CRP between both stages was − 3.4 mg/dl (IQR, − 1.2 to − 4.8 mg/dl) or 83% decrease (IQR 60–88%) and for IL-6 − 13 pg/ml (IQR, − 4 to − 20 pg/ml) or 64% decrease (IQR 32–80%).

10% (3/30) patients (3 male; 2 THAs and 1 TKA) were found to have persisting infection and had a minimum of two positive microbiological cultures intraoperatively (One patient *Staph. Epidermidis* in 3/5 samples, one patient with *Candida albicans* in 2/5 samples and one patient *Staph. Capitis* in 2/5 samples). In these cases, D-dimer level increased two times (1550 ng/ml to 2050 ng/ml and 2320 ng/ml to 2400 ng/ml) and decreased once (3910 ng/ml to 970 ng/ml). The serum values of CRP (5.5 mg/dl to 0.5 mg/dl, 1.3 mg/dl to 0.5 mg/dl and 12.1 mg/dl to 2.8 mg/dl) and IL-6 (28 pg/ml to 9 pg/ml, 10 pg/ml to 2 pg/ml and 25 pg/ml to 2 pg/ml) decreased in all 3 cases with persistent infection. Of these three cases, one patient died of unrelated cause (cardiovascular) three months postoperatively and the other two cases remain free from further surgical intervention and healed uneventfully with now 10 months of follow-up. The mean delta D-dimer level was 300 ng/ml (range − 2820 ng/ml to 4280 ng/ml) in eradicated infections, and − 380 ng/ml (range − 1720 ng/ml to 500 ng/ml) for persisting infections (*p* = 0.795).

Otherwise only one patient underwent revision for infection four months after reimplantation (female, 86 years, THA), and had a second two-stage exchange. In this case all three parameters decreased between both stages (D-dimer: 1.62 ng/ml to 1.09 ng/ml, CRP: 4.9 mg/dl to 0.5 mg/dl and IL-6: 29 pg/ml to 5 pg/ml).

## Discussion

The optimal timing of reimplantation surgery as part of a two-stage exchange for chronic PJI is difficult to determine and there is no individual marker to date that reliably indicates persisting infection. Therefore orthopedic surgeons often use a combination of inflammatory serum and synovial biomarkers^6,18^ to identify persisting infection, but still there is no gold standard to guide reimplantation^6^. Considering that the serum D-dimer has been included in the diagnostic criteria of the MSIS and ICM for the diagnosis of PJI, it appears valid to investigate this novel parameter and its change between stages for two-stage revision in order to potentially identify persisting infection. However, to our knowledge, only sporadic data has been reported for the use of serum D-dimer to identify persistent infection before reimplantation in two-stage exchange^14,15,19^. This study first systematically investigated the course of D-dimer in between stages in individual patients who underwent first stage explantation and second stage reimplantation. We found that serum D-dimers levels decreased in 50% (15 of 30 cases) and it increased in the other 50% questioning its usefulness as a marker that guides reimplantation timing or indicates infection persistence. In contrast, the level of CRP did not increase (0%; 0 of 30 cases) and the level of IL-6 increased only once in our patient collective (4%, 1 of 29 cases) with both marker showing a significant decrease in between stages.

The findings of this study need to be interpreted considering several limitations: Firstly, several factors that potentially influence D-dimers—such as different anticoagulants, different doses of anticoagulants, patient’s age and different microbiological organisms—are poorly understood in the setting of PJI and may have an impact on the course of D-dimers^19,20^. While future studies are needed to potentially establish adjusted D-dimer values, we believe that the effect of anticoagulation used could be limited in this study because of the uniform anticoagulation used during the study period. Secondly, the number of patients with persisting infection was limited and the persistence of infection is most likely multifactorial. Therefore, further studies with large number of patients are needed to confirm or refute our findings. Thirdly, the overall number of patients included was limited which makes it difficult to identify potential further confounding factors or conduct a useful calculation of a threshold for persisting infection that might have affected the results. Furthermore, the previous orthopedic literature on D-dimers has recently been criticized for basic methodological errors such as failure to report the specific laboratory standard used and the correct unit used with Moser et al.^21^ suggesting an individual cut-off for each institution as the levels of D-dimer are expected to vary greatly. This has been done for the present cohort^10^ however it remains uncertain whether it is useful for low-volume centers to gather a sufficient number of patients in order to calculate a reliable individual cut-off value. Lastly, it has been shown that some biomarkers and there behavior following surgery for PJI could predict further intervention or be associated with adverse outcome^22^. To our knowledge, this has not been investigated for D-dimer, but could be the subject of future studies that then should employ a minimum follow-up period. Due to the study design, we can only discuss potential revision surgeries for infection at short-term follow-up and otherwise relied on the primary healing of the wound and the absence of positive cultures during second-stage reimplantation to define infection control. It would be interesting to see if with longer follow-up there is a difference in reinfection rate depending on the change in D-dimers in between stages of the two-stage revision. However, due to the lack of a uniform decrease following first stage surgery, we remain skeptical whether D-dimer may be used for this purpose.

The serum level of D-dimer prior to reimplantation in two-stage exchange arthroplasty has been investigated in previous studies. Shahi et al.^14^ investigated 29 patients prior to reimplantation applying a calculated threshold of 850 ng/ml FEU from their cohort. While 2/5 patients with a D-dimer level above this threshold had positive cultures at the time of reimplantation and CRP and erythrocyte sedimentation rate were false negative in these cases, the other three patients were reported to have an uneventful staged reimplantation. Furthermore, the authors did not report D-dimer values at the time of first stage explantation surgery and spacer insertion for the individual patients. While we hypothesized that this change in between stages might be more helpful than using a strict threshold at the time of reimplantation, we found that 50% of patients had increasing levels of D-dimers while 50% had decreasing levels in between stages. Furthermore, the proposed cut-off of < 850 ng/ml prior to reimplantation was only met by 7% (2/30) patients in our cohort while a mere 57% (17/30) were below the cut-off that was calculated for our institution in a previous study questioning the usefulness of D-Dimers as a marker prior to reimplantation whatsoever.

Xu et al.^20^ report on the usefulness of D-dimers prior to reimplantation in 102 revision THA calculating a cut-off values of 820 ng/ml with all patients receiving rivaroxaban in the interim spacer period and found no difference in D-dimer levels between patients with persisting infection and with infection control. While the authors included a large number of patients, they did not collect D-dimer values prior to first stage surgery in their cohort, however in accordance to the present cohort, they also report poor performance in spite of a uniform use of anticoagulants in the interim spacer period mitigating this factor as a potential source of bias. One potential reason for the poor performance of D-dimer prior to reimplantation might be the occurrence of subclinical thromboembolic events in the interim spacer period as venous thromboembolism is a potential complication of two-stage exchange although we acknowledge the hypothetical nature of this idea^23^. However, no patient in the present cohort had thromboembolic events in the interim spacer period, nonetheless we believe that his notion should be investigated further.

While we found poor diagnostic accuracy using a definitive threshold for plasma D-Dimer levels as well as for the change of D-dimer levels in between stages, CRP and IL-6 showed a reliable and reproducible decrease in 93% and 96% of cases respectively. However, all parameters included in the study failed to identify the three patients with persisting infection as defined by the MSIS major criteria with > 2 positive cultures at reimplantation surgery. The absolute value of CRP as well as the ΔCRP have been studied before with fairly poor results regarding the sensitivity and specificity^6^. While Hoell et al.^17^ found a sensitivity of 42% and specificity of 84% in 106 two-stage revisions using an absolute value prior to reimplantation, while Ghanem et al.^24^ found a sensitivity between 29 and 71% and specificity of 15–73% depending on whether an absolute value or the ΔCRP was used in 109 two-stage revisions. On the other hand, Hoell et al.^7^ demonstrated that an absolute value of > 13 pg/ml of IL-6 was a reliable indicator of persisting infection which contradicts a more recent finding of Jiang et al.^25^ who investigated the change in Il-6 in between stages and concluded that it was a poor marker also given that the pre-reimplantation value of Il-6 was only 3 pg/ml in patients with reinfection. Considering that these studies all compared a similar two-stage exchange algorithm with an antibiotic holiday and an interval of around 6–8 weeks, it remains unclear whether these differences are patient or microorganism related and must be investigated in future adequately powered studies.

In conclusion, we do not recommend routine use of plasma D-dimer or the change of D-dimer levels between stages to guide the timing of reimplantation of a two-stage exchange arthroplasty. Future studies should try to identify potential factors that influence D-dimer levels during two-stage revision in order to improve the potential utility of this novel marker. Until then, surgeons must rely on a combination of more established parameters.
